# Targeting Lysosomal Dysfunction and Oxidative Stress in Age-Related Macular Degeneration

**DOI:** 10.3390/antiox14050596

**Published:** 2025-05-16

**Authors:** Ana S. Falcão, Margarida L. Pedro, Sandra Tenreiro, Miguel C. Seabra

**Affiliations:** iNOVA4Health, NOVA Medical School (NMS), Faculdade de Ciências Médicas (FCM), Universidade Nova de Lisboa, 1169-056 Lisboa, Portugal

**Keywords:** age-related macular degeneration, lysosomal dysfunction, NRF2, oxidative stress, retinal pigment epithelium, TFEB/mTORC1 axis

## Abstract

Age-related macular degeneration (AMD) is the leading cause of vision loss in the Western world, and it currently lacks effective therapy. It is believed that AMD initiates in the aged retinal pigment epithelium (RPE), which presents lysosomal dysfunction and oxidative stress (OxS) that ultimately leads to RPE damage and AMD progression. AMD is a complex pathology, so multitarget treatments are required to act on different pathways, presenting several challenges. In this review, we discuss the current knowledge on the pathogenesis of this disease, focusing mainly on lysosomal dysfunction and OxS. Because transcription factors regulate homeostasis, the transcription factor EB (TFEB), which controls lysosomal function and biogenesis, and the nuclear factor erythroid 2-related factor 2 (NRF2), which manages OxS, have been proposed as promising targets for disease intervention. Finally, we discuss the interplay of these pathways for a potential synergistic effect on AMD-targeted therapies, as they could change the course of today’s available treatments for AMD.

## 1. Introduction

Age-related macular degeneration (AMD) is a progressive retinal disorder that impacts millions worldwide, emerging as the primary cause of vision impairment in developed countries [1]. In fact, the predicted number of patients diagnosed with AMD has been growing significantly and is expected to reach 288 million in 2040 [2]. AMD is a multifactorial disease, so its genesis results from a combination of aging, environmental agents’ exposure, and genetic risk factors [3,4]. Since AMD-associated genes only support susceptibility, without proven causality [3], for the purpose of this review, only aging and environmental factors will be addressed. AMD is one of many age-related diseases. Most, if not all, chronic diseases develop when impaired cytoprotective pathways induce cell dysfunction severe enough to initiate tissue damage [5]. Determining the ways in which different endogenous and exogenous stressors described as hallmarks of aging (including telomere attrition, genomic instability, epigenetic changes, loss of proteostasis, deregulated nutrient sensing, cellular senescence, mitochondrial dysfunction, stem cell exhaustion, and altered intercellular communication) contribute to the regulation of aging is the main goal of current aging research [5]. Oxidative stress (OxS) and lysosomal dysfunction are two important and intertwined processes that greatly contribute to aging and related diseases. Lysosomal dysfunction impairs cellular cleanup, leading to the accumulation of damaged cellular components, while OxS stress accelerates cellular damage and inflammation [5,6]. When combined, these mechanisms produce a vicious cycle that speeds up aging and promotes the development of age-related illnesses.

**Figure 1:**
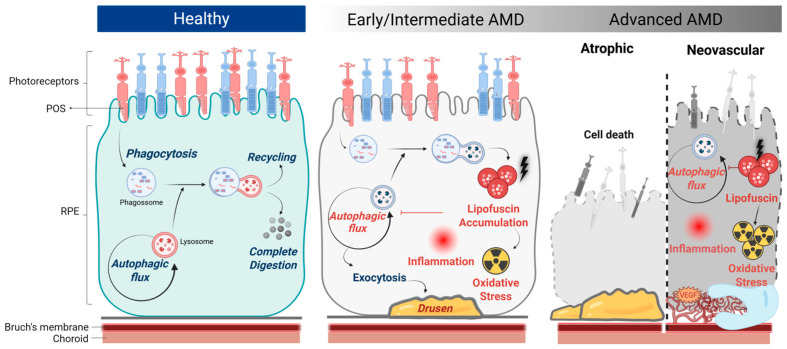
Retinal pigment epithelium (RPE) cellular changes through age-related macular degeneration (AMD) development. The healthy RPE is responsible for the daily digestion of photoreceptor outer segments (POS) by phagocytosis through its endo-lysosomal system which recycles some of its components and completely digests the remaining ones. In early/intermediate stages of AMD, exocytosis of this material may lead to progressive accumulation of biological “garbage,” and drusen on Bruch’s membrane, which contributes to oxidative stress buildup and inflammation. Ultimately, the disease can progress to the late stages of AMD, characterized by (i) atrophic AMD, when there is atrophy of photoreceptors, RPE, and choriocapillaris, and/or by (ii) neovascular AMD, when new vessels invade the RPE and eventually leak or rupture, resulting in fluid accumulation and/or hemorrhages. Created with BioRender (biorender.com/).

AMD primarily affects the central macular region of the human eye, involving the photoreceptors, retinal pigment epithelium (RPE), Bruch’s membrane, and the choroid [7]. The origin of AMD pathology appears to be the RPE. The RPE comprises phagocytic and pigmented cells with a polarized architecture and tight junctions in between cells that comprise part of the blood–retinal barrier [8]. The apical membrane of the RPE faces the photoreceptor outer segments (POS), where RPE’s long microvilli surrounds the POS, establishing a complex and close structural interaction. Meanwhile, the basolateral membrane of the RPE faces the Bruch’s membrane (Figure 1) [8,9]. The RPE facilitates multiple functions, including the transport of nutrients, ions, and water; light absorption and photo-oxidation protection; trans-retinal re-isomerization into 11-cis-retinal, which is crucial for the visual cycle; daily phagocytosis of shed POS; secretion of several factors essential for the structural integrity of the retina [8,9]; and the subretinal space ion composition stabilization, crucial for photoreceptor function [9]. As post-mitotic, terminally differentiated cells, these important functions place on them a unique burden over time, especially on their degradative machinery, and highlight the RPE’s susceptibility to damage [8,9]. The accumulation of functional deficits in RPE eventually leads to retinopathy as observed in Stargardt’s disease, retinitis pigmentosa, and AMD [9].

There is some consensus that AMD is initiated when aging and environmental stressors on the RPE trigger lysosomal dysfunction and an excess of reactive oxygen species (ROS) buildup, promoting the accumulation of autofluorescent undegradable materials, entitled lipofuscin, and chronic inflammation [1,10,11]. With time, the degradative machinery of the cell stops being able to respond to the lipofuscin accumulation, initiating a pathologic expansion to the extracellular space with the formation of drusen deposits. Early and intermediate AMD are characterized by medium/large drusen and fundus pigmentary abnormalities [2]. Advanced AMD involves loss of central vision, i.e., loss of photoreceptors and RPE, and can be classified into two main forms. The most prevalent, accounting for 85–90% of cases, is dry or atrophic AMD, which progresses slowly (over years) [4,10]. The atrophic form is often a risk factor for the more severe form, wet or neovascular AMD. This accounts for around 10–15% of all AMD cases, although geographic and demographic factors can influence the prevalence of these forms [12]. The neovascular form progresses more quickly (within days or months) and is characterized by choroidal vessels penetration of the Bruch’s membrane and RPE. Eventually, sub-retinal fluid accumulates and triggers macular edema, hemorrhage, and fibrosis. Ultimately, both forms culminate in permanent sight loss (Figure 1) [13].

With increased life expectancy, the world’s population ages with concomitant incidences of age-related diseases, like AMD. Unfortunately, currently available therapies only alleviate the disease’s physical complications and are unable to eliminate the pathology. There are currently few pharmacological therapeutic options for patients with neovascular AMD, consisting mainly of intraocular injections of vascular endothelial growth factor (VEGF) inhibitors. Unfortunately, many patients suffer from an incomplete response to anti-VEGF therapy [14] and because these do not address the underlying degeneration of AMD, the disease is simply delayed, and there is recurrence when treatment is discontinued [15,16]. The atrophic form of AMD, which constitutes the majority of cases, has no current treatment [15]. The difficulty in finding effective therapies to treat this disorder could be explained by the multitude of factors that lead to the disease phenotype. Hence, AMD therapeutics would benefit from a multifaceted approach [16]. Research has been directed towards managing early events in AMD to reveal new therapeutic targets, avoiding loss of RPE, which is critical for disease progression.

This review summarizes the current understanding of lysosomal dysfunction and OxS in RPE pathology and degeneration in the context of AMD. The known links between OxS and lysosomal pathways will be addressed, highlighting these mechanisms as potential targets for new therapeutic strategies in AMD.

## 2. Lysosomal Dysfunction in AMD

Lysosomes are central to numerous cellular functions, including endocytosis, phagocytosis, and autophagy. Despite their role in digestion and recycling, lysosomes also act as environmental sensors and play a pivotal role in several signaling pathways [17,18,19]. This aspect is particularly vital in the retina, which is prone to damage due to its high metabolic activity and exposure to photo-OxS [20]. Therefore, any dysfunction in lysosomal function or its communication with other organelles can significantly contribute to the onset and progression of major retinal degenerative disorders. In particular, lysosomes play a crucial role in autophagy, an internal “self-eating” mechanism responsible for the breakdown of damaged cellular components within autophagosomes, which merge with lysosomes for degradation. Given its high metabolic activity and status as a layer of post-mitotic cells, the RPE depends on autophagy to efficiently dispose of damaged macromolecules. Thus, compromised function or autophagy-lysosomal pathway dysfunction leads to a reduced autophagic flow, and subsequently threatens cellular health [17].

Photoreceptors are in constant and direct exposure to light. Over time, in these conditions, photonic damage can start affecting the POS neighboring proteins and lipids. For this reason, photoreceptors undergo continual turnover to avoid the buildup of oxidized components by the RPE. Thus, in addition to the bidirectional exchange of substances, the RPE is distinguished by being responsible for recognizing oxidized POS [21]. Even in healthy RPE, the accumulation of highly oxidized proteins (30–70%), lipids (20–50%), sugars, and metals (2%) originates lipofuscin [21,22] as a by-product of POS digestion [23,24]. With time and due to the daily burden of POS phagocytosis, the RPE accumulates lipofuscin granules. Given its polymeric and highly cross-linked nature, lipofuscin cannot be degraded, thus being accumulated within dysfunctional lysosomes and cell cytoplasm [22]. A potentially damaging effect of lipofuscin in RPE is an increased oxidation of molecules within its granules, leading to the formation of toxic products. These toxic products can cause lysosomal membrane permeabilization, inhibit lysosomal proton pumps or hydrolases, and diffuse outside the granules, causing damage to cellular organelles and cytoplasmic molecules [24].

The accumulation of lipofuscin and the subsequent increase in OxS within the cell induces stress conditions in lysosomes, mitochondria, and endoplasmic reticulum. This leads to the formation of misfolded protein aggregates and dysfunctional autophagy, which are associated with impaired protein clearance in RPE. With age, the accumulation of toxic lipid-protein aggregates may trigger inflammation, and the exocytosis of these materials will form extracellular drusen, a hallmark of AMD [25]. In fact, it was shown that autophagic and exosomal proteins are found in drusen, suggesting that increased autophagic activity, together with lysosomal dysfunction, may result in the release of intracellular proteins via exosomes [26]. Due to this impairment in lysosomal function, lysophagy has emerged as a new pharmacological target for AMD and other diseases involving lysosomal damage [27].

Recent research linked lysosomal dysfunction in AMD with chronic activation of the complement pathway in RPE [28]. Cerniauskas et al. showed that RPE cells containing the Y402H polymorphism in the complement factor H (*CFH*) gene, which significantly increases the risk of AMD, are characterized by a significant increase in the number of swollen lysosome-like vesicles with fragile membranes, cathepsin D leakage into drusen-like deposits, and reduced lysosomal function. The turnover of C3 is increased significantly in high-risk RPE cells, resulting in higher internalization and deposition of the terminal complement complex C5b-9 in lysosomes [28]. Lysosomes also play an important role in ion homeostasis, as the lysosome lumen is the storage site for several micronutrients, including calcium, sodium, zinc, copper, and iron [19]. In AMD, impaired lysosomal function can also result from iron overload in the RPE. Iron loading in these cells generates OxS, which causes lysosomal accumulation and dysfunction, resulting in ceramide buildup, lysosomal membrane permeabilization, and cell death [29]. Lysosomal calcium has several important regulatory roles, such as mediating fusion with other vesicular compartments, signal transduction, transcription factor regulation, lysosome reformation, autophagy, and lysosomal exocytosis [19]. Mice with calcium and integrin binding protein-2 deficiencies exhibit a phenotype similar to AMD, including sub-RPE deposits, a significant buildup of drusen indicators, impaired visual function, lysosomal membrane permeabilization, and cell death [30].

In summary, POS digestion by RPE cells places a constant and significant stress on these post-mitotic cells [8], triggering lipofuscin accumulation. Even though lipofuscin is naturally occurring and gradually accumulates with age [24], its accumulation is exacerbated by disease. Besides the RPE, lipofuscin granules are also known to accumulate in the heart, skeletal muscle, and neuronal cells, contributing to the development of neurodegenerative diseases [22]. Thus, future approaches targeting lipofuscin accumulation should be considered for these diseases. In this regard, remofuscin (a tetrahydropyridoether small molecule) was described to reverse lipofuscin accumulation in aged RPE cells and rescue retinal degeneration in a Stargardts disease mouse model [31]. Although the exact mechanism is yet unknown, remofuscin probably breaks down lipofuscin into small particles that are then likely eliminated via exocytosis [31]. This highlights the relevance of research into the processes that lead to lipofuscin accumulation and clearance.

## 3. Role of OxS in AMD

The macula possesses unique sources of ROS due to its high metabolic requirements. The macula is one of the most irrigated places of the human body, being exposed to an oxygen-rich environment [1,32,33], and is rich in mitochondria, which are a major source of ROS [1,34]. Also, high levels of ROS are produced in the RPE resulting from autophagy [35,36,37,38,39] and inflammation [40,41,42,43,44,45] dysregulation. Finally, the RPE daily digestion of POS is another major source of ROS, lipofuscin being one of its most reactive by-products, as described in the previous section. Exogenous sources also make their contribution to OxS, such as constant light exposure or cigarette smoking [33,46,47]. To counteract the harmful effects of ROS, the retina possesses several mechanisms of self-protection. Firstly, the retina holds a heavy antioxidant machinery of specific enzymes (such as cytochrome P450 mono-oxygenase system, superoxide dismutases (SOD), and catalases) as well as small molecular antioxidants (such as thiol, glutathione, and thioredoxin). These mechanisms are tightly regulated by several transcription factors, the most important of which is nuclear factor erythroid-2 related factor 2 (NRF2) that coordinates OxS defenses [33,46] (this topic will be discussed in more detail in Section 4.2). Moreover, chromophores in the retina, such as melanin, help protect against light-induced damage by absorbing excess light energy. The formation of melanolipofuscin granules in aged RPE, which contain both melanin and lipofuscin, has been linked to the development of AMD [46,47]. Finally, ROS stimulates RPE reparative autophagy, which protects RPE cells from oxidative damage and aims to restore damaged cellular structures [47].

The “redox stress hypothesis” states that the gradual breakdown of redox-regulated signaling systems is the main cause of age-related functional losses. Age-related changes in macromolecular state relate to a decrease in antioxidant defenses. The increase in ROS generation that is associated with aging is caused by an increase in the leakage of electrons from the electron transport chain. In addition, reduced adaptive induction of antioxidants and diminished antioxidant capacity have also been linked to aging [46]. AMD is thus manifested when the delicate balance between the physiological and pathological effects of ROS is disrupted, culminating in tissue damage [32]. An analysis conducted on the blood serum of AMD patients demonstrated increased levels of OxS markers, indicators of lipid peroxidation, protein oxidation, and oxidative DNA damage, when compared to healthy cohorts [48]. Hanus and colleagues showed that OxS induced by hydrogen peroxide (H_2_O_2_) on ARPE-19 cells leads to necroptosis [49]. Additionally, in a model of chronic OxS, human RPE cultures derived from adult stem cells exposed to tert-butylhydroperoxide (tBH) revealed upregulation of αB-, βB1-, βB2-, βS-, and βA4-crystallins, amyloid precursor protein, complement component 9, and VEGF-A, which are proteins commonly found in drusen [50]. Other AMD characteristics such as the development of drusen and RPE changes have been assessed in antioxidant and toxic response genes knockout mouse models for superoxide dismutase family (*Sod-1* and *2*), *Nrf2*, and aryl hydrocarbon receptor (*Ahr*, nuclear receptor that regulates cellular response to environmental signals, including UV and blue wavelength light) [51,52,53,54,55]. Furthermore, iPSC-RPE exposed to either H_2_O_2_ or tBH increased secretion of pro-angiogenic factors (VEGF, PTN, and CRYAB) and decreased secretion of anti-angiogenic factors (PEDF, CFH), which may play an important role in AMD pathophysiology [56]. Finally, injection of a strong inducer of acute OxS (NaIO_3_) in mice leads to RPE damage, including RPE atrophy, secondary photoreceptor damage, and retinal thinning [57,58,59,60,61]. Tools such as these allow a closer mimicking of the AMD pathology and support the idea that the ability to fight OxS by upregulating the antioxidant defense response mechanisms is possibly a key event in AMD initiation and progression.

## 4. Therapeutic Strategies Targeting OxS and Lysosomal Dysfunction in AMD

### 4.1. TFEB/mTORC1 Axis

The transcription factor EB (TFEB) is a member of the MiT-TFE helix–loop–helix leucine-zipper (bHLH-Zip) family of transcription factors and is described as a major controller of lysosomal function, biogenesis, and autophagy by positively regulating genes belonging to the coordinated lysosomal expression and regulation (CLEAR) network [62,63]. In diseases characterized by the buildup of toxic aggregates, such as lysosomal storage disorders, TFEB has been proposed as a key therapeutic target due to its role in modulating intracellular clearance pathways [64,65].

**Figure 2:**
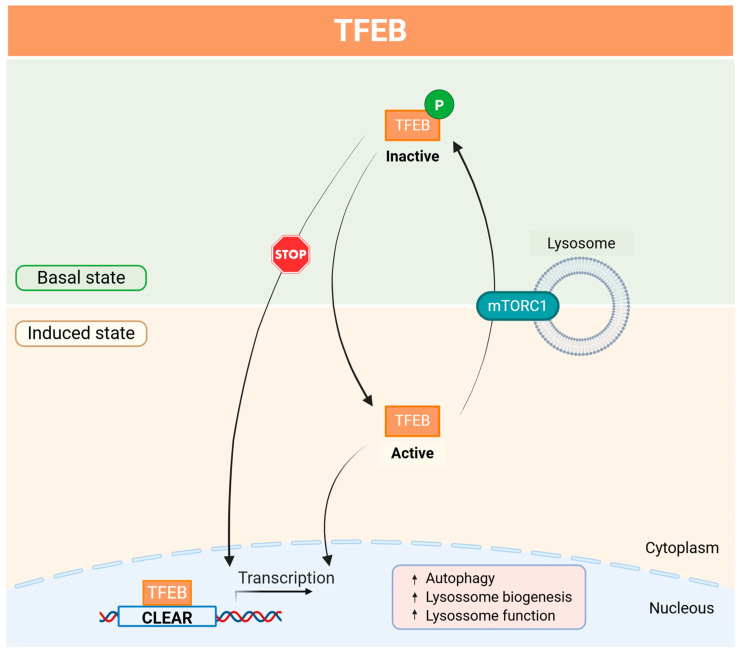
Molecular mechanism of the TFEB-mTORC1 pathway. The transcription factor EB (TFEB) is a master regulator of lysosome function, biogenesis, and autophagy. TFEB is sequestered in the cytosol by mechanistic target of rapamycin complex 1 (mTORC1)-mediated phosphorylation. When mTORC1 is inactive, such as under stress or nutrient starvation, TFEB phosphorylation is inhibited, leading to its nuclear translocation, where it activates the transcription of lysosomal and autophagy genes. CLEAR—coordinated lysosomal expression and regulation; mTORC1—mechanistic target of rapamycin complex 1; P—phosphorylation; TFEB—transcription factor EB. Created with BioRender (biorender.com/).

In cells heavily involved in lysosomal degradation, such as the RPE, the mechanistic target of rapamycin complex 1 (mTORC1) serves as the principal regulator of TFEB activity. Under nutrient-rich (anabolic) conditions, mTORC1 is activated at the lysosomal membrane, leading to the phosphorylation and inactivation of TFEB in the cytoplasm. In contrast, during stress or starvation (catabolic conditions), mTORC1 is inhibited, allowing dephosphorylated TFEB to translocate to the nucleus, where it initiates the transcriptional activation of target genes that regulate lysosomal biogenesis, function, and autophagy (Figure 2).

As lysosomal degradative function diminishes with age and disruptions in cellular clearance are associated with AMD development, TFEB emerges as a promising therapeutic target for addressing this disease. In fact, RPE from AMD donors showed abnormalities consistent with dysfunctional autophagy [66]. In RPE lysates from human AMD donors, an increased phosphorylation of TFEB was observed, which was not seen in age-matched non-AMD controls. In the same study, AMD patient’s eyes were studied, and they exhibited lower nuclear TFEB immunostaining than non-AMD control eyes. Cathepsin D and L protein levels and activities were also found to be lower in human AMD donor RPE [67]. Interestingly, cultured Cryba1 KO RPE cells (which have impaired lysosomal clearance) transduced with constitutively active TFEB-S210A increased mRNA expression of CLEAR network genes like *CTSB* (Cathepsin B), *LAMP2*, and *ATP6VOA1* (V-ATPase) [68]. Recently, we showed that overexpression of the constitutively active form of unphosphorylated TFEB dramatically reduces the accumulation of POS-dependent lipofuscin-like granules in a RPE in vitro model of AMD [69]. Also, in an in vitro AMD model using the cigarette oxidant hydroquinone (HQ), trehalose was cytoprotective against HQ-induced toxicity, upregulating autophagy markers and the autophagic flux [70]. Thus, stimulating autophagy or the lysosome pathway could increase the degradative capability of RPE cells.

The mammalian target of rapamycin (mTOR) signaling pathway, as the regulator of TFEB and possibly through other mechanisms, is also involved in the pathophysiology of AMD. In fact, in aged RPE cells, enhanced mTORC1 activity inhibits the degradation of photoreceptor outer segments (POS), potentially aggravating lysosomal dysfunction [71]. Thus, mTOR inhibitors have been recently considered as potential therapeutic strategy for this disease [69,72,73]. Rapamycin, a potent mTOR inhibitor, holds potential as a therapeutic option given its favorable safety profile and prolonged ocular pharmacokinetics. However, clinical trials have demonstrated limited efficacy and the occurrence of adverse effects (reviewed in [73]), which are restraining their use in humans. To overcome this issue, further research should focus on establishing the optimal low-dose range of rapamycin that effectively induces autophagy without causing retinal toxicity for the treatment of AMD. Specifically, longitudinal, controlled studies with larger, heterogeneous patient populations are necessary to determine the precise dosing and administration routes that balances efficacy and safety [74]. Nevertheless, in ARPE-19 cells exposed to A2E (a major toxic lipofuscin component linked to AMD), rapamycin induced autophagy through mTOR inhibition, leading to a reduction in A2E accumulation and a decrease in the protein expression of inflammation-associated and angiogenic factors [75]. We showed that treatment of POS-fed cells with rapamycin after the appearance of lipofuscin-like autofluorescent granules results in a reduction in these granules. This effect is dependent on active lysosomal enzymes and induction of active dephosphorylated TFEB [69]. Recent studies suggest that synthetic high-density lipoprotein nanoparticles delivering rapamycin may offer a more effective therapeutic delivery method for treating AMD [76]. Several derivatives of rapamycin (rapalogs) have been developed, with improved pharmacokinetics [77], which can also emerge as an attractive AMD treatment.

Therefore, understanding the role of the TFEB/mTOR in the mechanisms of phagocytosis and clearance of POS could help identify specific molecular targets for the development of more effective drugs to prevent or reduce the formation and accumulation of lipofuscin. New drug delivery techniques and gene therapy, as well as selective regulators in the mTOR pathway, could reduce adverse effects and offer more precise treatments, ultimately improving therapeutic outcomes [73].

### 4.2. NRF2/NFE2L2

**Figure 3:**
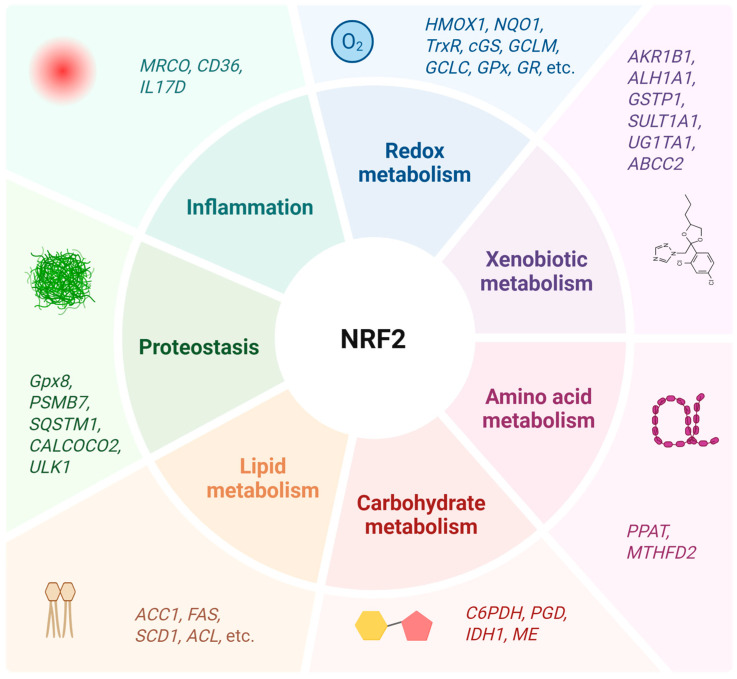
NRF2, a master regulator of cell homeostasis. The nuclear factor erythroid-2 related factor 2 (NRF2) regulates more than 250 genes involved in antioxidant response, xenobiotic, amino acid carbohydrate, and lipid metabolism, inflammation, and proteostasis balance. *ABCC2*—ATP-binding cassette subfamily C member 2; *ACL*—ATP citrate lyase; *ACC1*—acetyl-coenzyme A carboxylase 1; *AKR1B1*—aldo-keto reductase family 1B member 1; *ALH1A1*—aldehyde dehydrogenase family 1A member 1; *CALCOCO2*—calcium-binding and coiled-coil domain 2; *cGS*—c-glutamate cysteine synthetase; *FAS*—fatty acid synthase; *G6PDH*—glucose-6-phosphatedehydrogenase; *GPx*—glutathione peroxidase; *Gpx8*—glutathione peroxidase 8; *GR*—glutathione reductase; *GSTP1*—glutathione peroxidase P1; *HMOX1*—heme oxygenase-1; *IDH1*—isocitratedehydrogenase 1; *ME*—malic enzyme; *MTHFD2*—methylenetetrahydrofolate dehydrogenase 2; *PGD*—phosphogluconate dehydrogenase; *PPAT*—phosphoribosyl pyrophosphate amidotransferase; *PSMB7*—proteasome subunitbtype-7; *SCD1*—stearoyl-CoA desaturase; *SULT1A1*—sulfotransferase family 1A member 1; *TrxR*—thioredoxin reductase; *UG1TA1*—UDP glucuronosyltransferase family 1 member A1; *ULK1*—unc-51-like autophagy-activating kinase 1. Adapted from [89]. Created with BioRender (biorender.com/).

NRF2 transcription factor encoded by the NFE2-Like BZIP transcription factor-2 (*NFE2L2*) gene, is a member of the “leucine zipper” family of cap ‘n’ collar (CNC) factors that bind DNA by forming homo- or heterodimers [78,79,80,81]. NRF2 controls gene transcription by binding to antioxidant response elements (ARE) (core sequence 5′-A/GTGAC/TnnnGCA/G-3′) [80,82,83] present in the promotor regions of about 250 genes involved in antioxidant response, mitochondrial function, detoxification, proteostasis, and inflammation [13,79,80,84,85]. Other metabolic actions include lipid, carbohydrate, nucleotide, and amino acid metabolism [79,81,85] (Figure 3).

**Figure 4:**
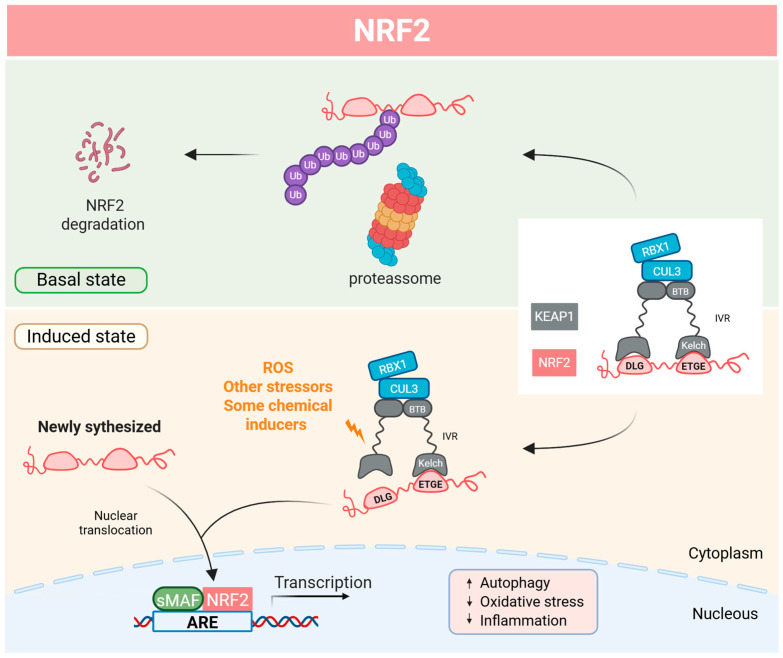
Molecular mechanism of the NRF2-KEAP1 pathway. Under basal conditions, the Kelch-like ECH-associated protein 1 (KEAP1) drives the nuclear factor erythroid-2 related factor 2 (NRF2) to ubiquitination by the CUL3-RBX1 and consequent degradation by the 26S proteasome. Under induced conditions, such as in the presence of reactive oxygen species and other stressors, KEAP1 is inactivated, preventing NRF2 degradation; then, together with the NRF2 de novo synthesize, NRF2 is accumulated and translocated into the nucleus where it dimerizes with sMAF and binds to ARE-sequences, activating the expression of antioxidant, detoxification, anti-inflammatory, and autophagy genes. ARE—antioxidant response element; BTB—bric-a-brac domain; CUL3—cullin 3; IVR—intervening region; RBX1—RING-box protein 1; ROS—reactive oxygen species; sMAF—small musculoaponeurotic fibrosarcoma oncogene homologue; Ub—ubiquitin. Adapted from [86]. Created with BioRender (biorender.com/).

The most relevant NRF2 regulator at the level of protein stability is Kelch-like ECH-associated protein 1 (KEAP1). KEAP1 is a dimeric protein and a redox sensor of the antioxidant response, as it possesses several highly reactive cysteines [81,86]. Additionally, this protein belongs to the Broad complex/Tramtrack/Bric-a-brac (BTB)-Kelch family, which is known to assemble Cullin 3 (herein CUL3) and RBX1 to form multisubunit Cullin–Really Interesting New Gene (RING)-ligases (CRLs) for protein ubiquitination. Due to interaction with KEAP1, NRF2 is a ubiquitously expressed protein with a high turnover and half-life of about 20 min [86,87,88]. Under basal redox conditions, NRF2 protein levels are low due to cytoplasmic complexation with KEAP1, which directs it for proteasome degradation. The KEAP1 homodimer binds, through the Kelch domain, to the low and high-affinity motifs (DLG and ETGE, respectively) of NRF2, thus mediating its ubiquitination by the CUL3-RBX1 complex, which is assembled at the BTB domain of KEAP1. Once ubiquitinated, NRF2 is rapidly recognized and degraded by the 26S proteosome. However, stressors such as OxS or some electrophilic compounds like xenobiotics react with cysteines of KEAP1, resulting in protein modifications that inactivate KEAP1-binding to NRF2. Because the turnover of NRF2 is faster than KEAP1, newly synthesized NRF2 does not have free KEAP1 to bind and is translocated into the nucleus. Once in the nucleus, NRF2 dimerizes with sMAF and the complex binds to ARE sequences, promoting the expression of ARE genes (Figure 4) [13,85,86,88,89,90].

The NRF2 gene is highly polymorphic, with a mutagenic frequency of 1 per every 72 bp [89,91]. Several somatic mutations and single-nucleotide polymorphisms (SNPs) have been identified for the *NFE2L2* [91,92]. Reported somatic mutations fall within the KEAP1 binding domain, which is the most important player of NRF2 regulation. Similarly, most of the reported SNPs are located in the noncoding regions of the gene and are thus presumably involved in *NFE2L2* gene regulation. Hence, a number of these SNPs may set functional haplotypes and be linked to the risk of developing chronic diseases or even be used as therapeutic targets [89,91]. Importantly, a single-nucleotide polymorphism in the NRF2 encoding gene was associated with AMD risk [93]. Therefore, genetic studies comparing healthy and patient cohorts are crucial to evaluate the correlation with NRF2 polymorphisms. These studies would also be crucial to optimize NRF2 therapies, since it is still unknown how populational NRF2 activity variation can impact the effectiveness of these therapies [94].

In general, NRF2 is considered a master regulator of cell homeostasis, and its action on a wide range of physiological processes suggests NRF2 as a promising target for the development of novel treatments for a variety of diseases, including neurodegenerative, autoimmune, metabolic, cardiovascular, and cancer. In fact, its multitarget feature makes it an interesting and necessary tool for testing innovative therapies for chronic diseases with a complex network of pathogenic pathways [86]. Age-related diseases such as AMD appear to be particularly suited for NRF2 modulation since there is evidence that NRF2 activity declines with aging [85,95] and that there is NRF2 involvement in most of the hallmarks of aging [84]. For example, NRF2 signaling is impaired in RPE in aged mice [96]. Furthermore, *Nrf2^−/−^* mice develop AMD-like phenotypes, such as drusen-like deposits, increased autofluorescence, and sub-RPE deposition of inflammatory proteins [52]. Interestingly, the RPE of *Nrf2^−/−^* mice also accumulated large amounts of autophagosomes and autolysosomes, suggesting impaired macroautophagy [52]. These phenotypes are exacerbated by exposure of *Nrf2^−/−^* mice to mild white light [97].

Some studies have highlighted the therapeutic potential of Adeno-associated virus (AAV)-NRF2 in retinal disease and AMD. A study evaluating the antioxidant and anti-inflammatory properties of AAV delivering an NRF2 peptide demonstrated its ability to induce antioxidant gene expression, protect RPE from oxidative injury, and photoreceptor function in a mouse model of oxidative injury with sodium iodate intravitreal injection [98]. Another study demonstrated that AAV-*Nrf2* promotes structure preservation and retinal functiona recovery in a light-induced mouse model of AMD [99].

Given its importance in many physiological processes and its potential utility as a therapeutic target in several disorders, a variety of drugs/NRF2 inducers are under development. Targeting KEAP1 inactivation or disrupting the KEAP1-NRF2 interaction offers a promising strategy for pharmacological activation of NRF2. Dimethyl fumarate (DMF) is a notable example already approved in the USA and Europe for the treatment of psoriasis and relapsing-remitting multiple sclerosis. DMF acts as a pro-drug with its derived metabolite, monomethylfumarate (MMF), which rapidly interacts with cysteine-151 in the KEAP1 BTB domain to activate the NRF2 pathway. This is not, however, its only mechanism of action, since it can also interact with NRF2-independent pathways such as the pro-inflammatory NF-kB pathway [86,94]. In fact, the anti-inflammatory effects of most electrophilic NRF2 activators are thought to be at least partly NRF2-independent, suggesting that these compounds lacking specificity may be advantageous for multitargeted pathologies [94]. As an NRF2 activator, DMF could be used to treat other pathologies. Some studies have already revealed that DMF has protective capacities after light-induced retinal damage [100,101]. Thus, DMF remains an investigational therapy for AMD and long-term randomized controlled trials are necessary to confirm its potential benefit and to establish standardized dosing regimens.

Several other studies have highlighted the ability of distinct (poly)phenols, such as curcumin, sulforaphane (SFN), and resveratrol to mitigate OxS-induced damages through activation of the NRF2 pathway and target AMD pathology. Curcumin was demonstrated to regulate the proliferation, OxS, and apoptosis of RPE cells [102,103,104] and there is an ongoing clinical trial to study the effect of oral curcumin supplementation in AMD patients (clinicalTrials.gov: NCT04590196). In vitro and in vivo data demonstrate that SFN maintains retinal function and morphological integrity and preserves cone function in a Nrf2-dependent manner [105,106,107]. Current clinical studies suggest that resveratrol supplementation may offer adjunctive benefits in the management of wet AMD, including reduced need for intravitreal injections, decreased progression of macular fibrosis, and improvements in patients’ quality of life [108]. More studies are currently being conducted to assess the safety and efficacy of these and other natural compounds as AMD therapies.

## 5. Interplay Between the TFEB/mTOR and NRF2/KEAP1 Pathways for a Synergistic Effect on AMD-Targeted Therapies

Throughout this review, we promote the idea that AMD is a chronic disease with complex pathological pathways, which would benefit from a multitargeted approach against lysosome dysfunction and OxS. Recent studies have proposed the existence of bridging factors connecting the TFEB/mTOR and NRF2/KEAP1 pathways. The crosstalk between these pathways could pave the way for novel dual-targeted therapies for AMD. To date, it is still unclear whether it is aging-driven lysosomal dysfunction and/or lipofuscin accumulation that induces increased OxS and consequent RPE damage, or whether OxS is the primary contributor for AMD pathology by disrupting lysosomal function and subsequently impairing the lysosomal clearance of autophagy cargo and phagocytosed material. Thus, it is important to better understand the molecular mechanisms involved and how targeting these pathways could ameliorate cellular stress and protein homeostasis in AMD.

TFEB can activate NRF2 under conditions devoid of OxS [109]. In a human cell line stably expressing TFEB, NRF2 activity is strongly enhanced through its stabilization due to repression of the NRF2-specific E3 ubiquitin ligase, DCAF11. In addition, the level of phosphorylated p62 was highly increased, which may interfere with the association of KEAP1 and NRF2, thus stabilizing NRF2 [109]. This p62-dependent regulation of NRF2, also known as non-canonical p62-NRF2 activation, regulates NRF2 activity through the autophagic degradation of KEAP1. This process is mediated by p62, a selective autophagy cargo receptor, which facilitates KEAP1 sequestration into autophagosomes. This occurs via a direct interaction between p62 STGE motif and the KEAP1 Kelch domain. Through competitive binding for KEAP1, p62 interferes with NRF2 ubiquitination, leading to prolonged NRF2 stabilization and activation [110]. These findings suggest that targeting TFEB could have a dual therapeutic effect in activating both NRF2 and the autophagy/lysosome pathway. In line with these findings, NRF2 was identified as a target gene of TFEB, where TFEB can regulate redox homeostasis via NRF2 [111,112]. A good example of a drug with a mechanism of action that bridges both transcription factors is SFN, an electrophilic compound found in cruciferous vegetables such as broccoli, and a powerful inducer of cellular antioxidant responses [112]. SFN induces TFEB nuclear translocation by stimulating a moderate increase in ROS, which in turn dephosphorylates TFEB via a Ca^2+^-calcineurin-dependent but mTOR-independent mechanism. The expression of genes required for lysosome biogenesis is subsequently increased by activated TFEB, which facilitates the clearance of damaged mitochondria. Interestingly, TFEB activity seems to be required for SFN-induced NRF2 activation to protect against OxS [111]. Therefore, this drug may initiate a self-defense cellular process that can successfully reduce OxS, which is frequently linked to a number of metabolic and age-related disorders, by concurrently activating autophagy and detoxification pathways. The protective effects and key mechanisms of action of SFN appear to be relevant in AMD, as supported by in vitro and in vivo data (reviewed in [107]).

Conversely, the NRF2/KEAP1 pathway can also activate TFEB-dependent lysosomal biogenesis and play a critical role in the maintenance of lysosomal homeostasis during embryonic development [113]. This is consistent with the previous finding that loss of KEAP1 induces lysosomal biogenesis independently of mTORC1, affecting cathepsin D activity and promoting the accumulation of non-degradative organelles [114]. Loss of NRF2 was also found to decrease TFEB nuclear translocation and TFEB-dependent transcription of the lysosomal protein VAMP8, supporting a role for NRF2 in TFEB regulation [115]. It was shown that NRF2 indirectly controls VAMP8 through the TFEB/mTOR axis. In NRF2 KO cells, a decrease in TFEB-dependent transcription of VAMP8 results in blockage of autophagosome-lysosome fusion and inhibition of ferritinophagy, implicating the NRF2-TFEB-VAMP8 axis in iron homeostasis [113]. Activation of this signaling network could represent an additional benefit in AMD, as iron is implicated in AMD pathogenesis [116], as already discussed in Section 2.

NRF2 is a key regulator of the autophagy–lysosome pathway, orchestrating the expression of several essential autophagy-related genes, including *ULK1*, *ATG5*, *ATG7*, and *SQSTM1*/p62 [110,117]. These genes play pivotal roles in autophagosome formation and the degradation of cellular cargo, ensuring the effective removal of damaged organelles and protein aggregates—a process crucial for maintaining cellular integrity and homeostasis. This NRF2-dependent control facilitates the removal of damaged organelles and misfolded proteins, promoting cellular stability. Beyond macroautophagy, NRF2 also supports chaperone-mediated autophagy by promoting the transcription of *LAMP2A*, a lysosomal receptor vital for this selective degradation pathway [117]. Notably, the deletion of ATG5 and ATG7 results in prolonged NRF2 activation through a non-canonical, p62-dependent route [110,118]. Thus, this intricate interaction between NRF2 and the protein degradation machinery is vital for preserving cellular balance under stress, enhancing survival by reducing proteotoxic and oxidative damage.

**Figure 5:**
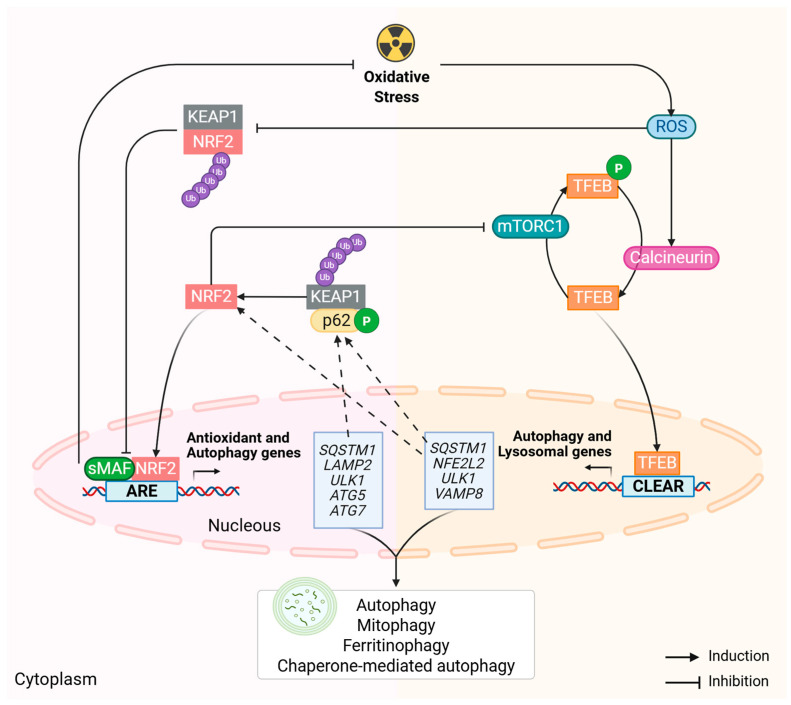
Linking factors that integrate the TFEB-mTOR and NRF2-KEAP1 pathways. The transcription factor EB (TFEB) acts by inducing autophagy and lysosome biogenesis, while the nuclear factor erythroid-2 related factor 2 (NRF2) induces autophagy and antioxidant enzymes. Bridging factors have been identified, but there are still mechanisms that remain unknown. AMD therapies would strongly benefit from the target combination of both pathways. Dashed lines highlight NRF2 and TFEB downstream targets directly participating in the TFEB and NRF2 signaling pathways, respectively. *ATG5*—autophagy-related 5; *ATG7*—autophagy-related 7; ARE—antioxidant response element; CLEAR—coordinated lysosomal expression and regulation; KEAP1—Kelch-like ECH-associated protein 1; *LAMP2*—lysosomal-associated membrane protein 2; mTORC1—mechanistic target of rapamycin complex 1; *NFE2L2*—nuclear factor erythroid-2-related factor 2; P—phosphorylation; *SQSTM1*/p62—sequestosome 1; ROS—reactive oxygen species; sMAF—small musculoaponeurotic fibrosarcoma oncogene homologue; Ub—ubiquitin; *ULK1*—Unc-51-like autophagy-activating kinase 1; *VAMP8*—vesicular-associated membrane protein 8. Created with BioRender (biorender.com/).

Therapeutic approaches that improve lysosomal biogenesis may rejuvenate lysosomal function, providing neuroprotection to the retina [17]. Recently, highly conserved ARE-linked sequences located in noncoding regions of the human *TFEB* and mouse *Tfeb* genes have been found, and exposure to KEAP1/NRF2/ARE system activators leads to a dose-dependent induction of TFEB-related genes, as well as a gradual increase in the number of lysosomes and the intensity of autophagosome–lysosome fusion [119]. These observations reinforce the important role of the TFEB/mTOR and NRF2/KEAP1 pathways as novel dual-targeted therapies against AMD. Future studies are needed to fully understand the intricate feedback loops between these pathways (Figure 5). Importantly, both TFEB and NRF2 need to be carefully modulated. NRF2 constitutive activation occurs in a variety of cancers, and aberrant NRF2 activation is being correlated with cancer progression, chemoresistance, and radioresistance [120]. Similarly, TFEB constitutive activation leads to renal carcinomas [121]. Thus, in addition to better understanding of the crosstalk between these pathways in AMD, we also require refinement of this knowledge to cell-specific targeted therapies.

## 6. Conclusions

AMD progression from intermediate to late AMD leads to a point of irreversible RPE degeneration where treatment becomes worthless. Treating patients at the early/intermediate stages presents a better therapeutic window opportunity for AMD as the disease could potentially be prevented or slowed down. Strong evidence points to RPE dysfunction at these stages, mainly through redox imbalance and lysosomal dysfunction in RPE oxidative injury. Thus, restoring oxidative balance and lysosomal function may act as preventive and therapeutic measures against RPE dysfunction and degeneration. The key transcriptional regulatory factors of related pathways, such as TFEB and NRF2, may be targeted to restore homeostasis and/or prevent further RPE degeneration. However, studies for novel dual-targeted therapies are still at an early stage, and several challenges limit their clinical application. Systemic activation risks toxicity and off-target effects, particularly in elderly patients, while poor tissue specificity and inefficient delivery hinder precise modulation in the retina. Intracellular delivery to RPE cells remains difficult, and the long-term ocular effects of sustained TFEB-NRF2 activation are not fully understood. Furthermore, their therapeutic impact may vary depending on the stage of AMD. Potential solutions include retina-specific gene delivery using AAV vectors, localized administration (e.g., intravitreal injection), nanoparticle or exosome-based delivery systems, and inducible or cell-specific promoters to enhance safety and precision. Stratifying patients by disease stage and using retinal imaging and molecular biomarkers can help tailor and monitor such interventions more effectively. Thus, dual-targeting of TFEB and NRF2 offers a promising therapeutic strategy for AMD, and future research should focus on elucidating their interplay while advancing retina-specific delivery systems, stage-tailored interventions, and robust preclinical models to unlock their full clinical potential.

## Abbreviations

The following abbreviations are used in this manuscript:

AAV	Adeno-associated virus
Ahr	aryl hydrocarbon receptor
ABCC2	ATP-binding cassette subfamily C member 2
ACl	ATP citrate lyase
ACC1	acetyl-coenzyme A carboxylase 1
AKR1B1	aldo-keto reductase family 1B member 1
ALH1A1	aldehyde dehydrogenase family 1A member 1
AMD	age-related macular degeneration
ATG5	autophagy-related 5
ATG7	autophagy-related 7
ATP6VOA1	V-ATPase
ARE	antioxidant response element
BTB	bric-a-brac domain
CALCOCO2	calcium-binding and coiled-coil domain 2
CFH	complement factor H
cGS	c-glutamate cysteine synthetase; FAS, fatty acid synthase
CLEAR	coordinated lysosomal expression and regulation
CNC	cap ‘n’ collar
CRL	cullin–RING ligases
CRYAB	Alpha-B crystallin
CTSB	cathepsin B
CUL3	cullin 3
DMF	dimethyl fumarate
G6PDH	glucose-6-phosphatedehydrogenase
GPx	glutathione peroxidase
Gpx8	glutathione peroxidase 8
GR	glutathione reductase
GSTP1	glutathione peroxidase P1
HMOX1	heme oxygenase-1
HQ	hydroquinone
IDH1	isocitratedehydrogenase 1
IVR	intervening region
KEAP1	Kelch-like ECH-associated protein 1
LAMP2	lysosomal-associated membrane protein 2
ME	malic enzyme
MMF	monomethylfumarate
MTHFD2	methylenetetrahydrofolate dehydrogenase 2
mTOR	mammalian target of rapamycin
mTORC1	mechanistic target of rapamycin complex 1
NFE2L2	NFE2-Like BZIP transcription factor-2
NF-kB	nuclear factor-κB
NRF2	nuclear factor erythroid-2 related factor 2
OxS	oxidative stress
PEDF	pigment epithelium-derived factor
PGD	phosphogluconate dehydrogenase
POS	photoreceptor outer segments
PPAT	phosphoribosyl pyrophosphate amidotransferase
PSMB7	proteasome subunitbtype-7
PTN	pleiotrophin
p62	sequestosome 1
RPE	retinal pigmented epithelium
RBX1	RING-box protein 1
ROS	reactive oxygen species
SCD1	stearoyl-CoA desaturase
SFN	sulforaphane
SNP	single-nucleotide polymorphism
SOD	superoxide dismutases
SQSTM1	sequestosome 1
SULT1A1	sulfotransferase family 1A member 1
sMAF	small musculoaponeurotic fibrosarcoma oncogene homologue
tBH	tert-butylhydroperoxide
TFEB	transcription factor EB
TrxR	thioredoxin reductase
UGT1A1	UDP glucuronosyltransferase family 1 member A1
ULK1	Unc-51-like autophagy-activating kinase 1
VAMP8	vesicular associated membrane protein 8
VEGF	vascular endothelial growth factor
